# hsa‐mir‐133a‐2 promotes the proliferation and invasion of cervical cancer cells by targeting the LAMB3‐mediated PI3K/ATK pathway

**DOI:** 10.1002/cam4.5380

**Published:** 2022-10-28

**Authors:** Xiaoyu Sui, Yurong Sun, Guiyu Zhang, Na Chi, Yitong Guan, Dan Wang, Xiulan Li

**Affiliations:** ^1^ Department of Obstetrics and Gynecology The First Affiliated Hospital of Qiqihar Medical University Qiqihar P. R. China; ^2^ Teaching and Research Section of Pathology Qiqihar Medical University Qiqihar P. R. China; ^3^ Department of Obstetrics and Gynecology Qilu Hospital of Shandong University Shandong P. R. China

**Keywords:** cell proliferation, cervical cancer, hsa‐mir‐133a‐2, LAMB3, PI3K/AKT signaling pathway

## Abstract

**Objective:**

Cervical cancer, one of the common types of malignant tumors progressed in women, is on the rise in developing countries. Numerous previous studies have demonstrated that hsa‐mir‐133a‐2 miRNA is abnormally expressed in cervical cancer cells. However, its fundamental mechanism in cervical cancer needs to be further clarified. Our study set out to investigate the effect of hsa‐mir‐133a‐2 on the phenotypes of cervical cancer cells as well as any potential molecular processes involved in the proliferation and invasion of cervical cancer cells.

**Methods:**

The Cancer Genome Atlas‐cervical squamous cell carcinoma and endocervical adenocarcinoma(TCGA‐CESC) was adopted in order to verify the expression of hsa‐mir‐133a‐2 in cervical cancer tissues and to identify its potential targets. The interaction between Laminin subunit beta‐3(LAMB3) and hsa‐mir‐133a‐2 was verified by TargetScan database as well as Luciferase reporter assay. The Cell Counting Kit‐8 (CCK8) and transwell methods were utilized to assess the influence of hsa‐mir‐133a‐2 on the proliferation and invasion characteristics of cervical cancer cells. We studied the role that hsa‐mir‐133a‐2 plays in cervical cancer progression through Kyoto Encyclopedia of Genes and Genomes(KEGG) analysis as well as Western Blot (WB) experiment.

**Results:**

Down‐regulation of hsa‐mir‐133a‐2 was detected in cervical cancer tissues. It directly targeted LAMB3 and negatively regulated LAMB3 expression. The overexpression of hsa‐mir‐133a‐2 has a significant inhibiting effect on cervical cancer cell proliferation and invasion. The overexpression of hsa‐mir‐133a‐2 significantly inhibits the proliferation and invasion of cervical cancer cells. Moreover, the LAMB3 was able to up‐regulate the phosphorylation levels of AKT and phosphatidylinositol 3‐kinase (PI3K) protein in cervical cancer cells. hsa‐mir‐133a‐2 could also modulate the PI3K/AKT signaling pathway by targeting LAMB3.

**Conclusion:**

hsa‐mir‐133a‐2 inhibits cervical cancer cell proliferation and invasion by indirectly regulating the PI3K/AKT signaling pathway, providing us with a new clinical treatment strategy for cervical cancer.

## Graphical abstract

This study found that hsa‐mir‐133a‐2 directly targets LAMB3 and negatively regulates its expression in cervical cancer. Hsa‐mir‐133a‐2 inhibits PI3K / Akt pathway by targeting LAMB3, and then inhibits the proliferation and invasion of cervical cancer cells.

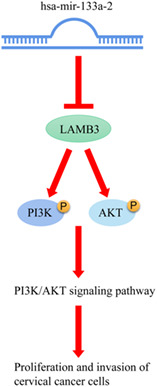

## 1 INTRODUCTION

Cervical cancer, one of the prominent type of malignant tumors found in women, poses a major threat to their health. In 2020, there were approximately 604,000 new incidences of cervical cancer and 342,000 deaths as a result of the disease worldwide.^1^ When most patients are diagnosed with cervical cancer, they are in the middle or advanced stages, and conventional treatment has limited effect. The five‐year survival rate for metastatic cervical cancer is merely 16.5%. Meanwhile, the median survival period is 8–13 months.^2^, ^3^ Most areas in China lack effective early screening and treatment protocols for cervical cancer. Thus, the morbidity as well as mortality rate resulting from cervical cancer is lacking effective control.^4^, ^5^ Therefore, we found it was critical to explore the underlying molecular mechanisms that triggered cervical cancer progression, and to promote the establishment of new diagnostic biomarkers for cervical cancer, and to screen potential therapeutic targets.

MicroRNA (miRNA) is a form of endogenous non‐coding small RNA that contains 21–23 nucleotides. A number of preliminary investigations have discovered that those miRNAs are dysregulated in various forms of cancers. They also play a role in the development and progression of many types of cancers. It is essential for cellular differentiation, cell proliferation, apoptosis as well as cell migration.^6^ A vast number of miRNAs have been shown to have a triggering or inhibiting effect on cervical cancer, and are likely to be adopted as therapeutic targets therapeutically or diagnostic biomarkers during the cervical cancer therapy process. For example, DNA methylation of miR‐138 regulates cell proliferation and epithelial—mesenchymal transition(EMT) in cervical cancer by targeting EZH2.^7^ MiR‐599, which is significantly under‐expressed in cervical cancer, suppresses cell proliferation, migration, and invasion.^8^

hsa‐mir‐133a‐2 is located on chromosome number 20. Numerous studies have demonstrated that it is abnormally expressed in solid tumors such as colorectal cancer, breast cancer and prostate cancer, and that it is closely associated to tumor patient prognosis.^9^, ^10^, ^11^ In addition, hsa‐mir‐133a‐2 was also found to be involved in the regulation of tumor cell proliferation, migration, invasion, and drug resistance, indicating its significant potential as a tumor therapeutic target.^12^ Yuan et al. reported that miR‐133a can act as a sponge miRNA and bind to competing endogenous RNAs NEAT1 and SOX4 to promote and participate in the development of cervical cancer.^13^ However, other roles played by hsa‐mir‐133a‐2 in cervical cancer pathogenesis remain unclear. Therefore, the purpose of our study is to discover the potential hsa‐mir‐133a‐2 molecular mechanism during the progression of cervical cancer.

Laminin subunit beta‐3 (LAMB3) is one of the important subunits that participated in laminin. Numbers of related studies have demonstrated that LAMB3 played a vital role in the invasion and metastasis of malignant tumor cells such as pancreatic cancer and lung cancer.^14^, ^15^ LAMB3 was involved in the coding of laminin‐5 (LN‐5) in the cervix. And LN‐5 is related to cervical lesions. As a result, the expression level of LAMB3 in patients can also reflect the early stage of cervical lesions to a certain extent.^16^ In addition, Yamamoto et al. discovered that LAMB3, as a target of miR‐218, is involved in regulating the migration and invasion of cervical cancer cells.^17^ In this study, functional enrichment analysis was employed to explore other molecular regulatory mechanisms of hsa‐mir‐133a‐2 during cervical cancer development and to verify such a mechanism at the cellular level.

The differential gene analysis of The Cancer Genome Atlas‐cervical squamous cell carcinoma and endocervical adenocarcinoma(TCGA‐ECSC), as well as the results of cell experiments, demonstrated that there is a significant decrease in expression of hsa‐mir‐133a‐2 found within cervical cancer tissues as well as cells. However, its regulatory mechanism in cervical cancer cells remains unknown. Therefore, our research means to explore the role of hsa‐mir‐133a‐2 plays on the proliferation as well as invasion of cervical cancer cells. We also would like to explore its underlying molecular mechanisms.

## 2 METHODS

### 2.1 TCGA data collection and analysis

The transcriptome data (RNA‐Seq) and related clinical information of cervical cancer (cervical squamous cell carcinoma, CESC) patients in TCGA was obtained through the genome data sharing portal, namely GDC portal (https://portal.gdc.cancer.gov/). We integrated transcriptome data in TCGA‐CESC utilizing R software and extracted the miRNA and messenger RNA(mRNA) expression profiles separately. The “edge” package of R software was employed to perform differential gene analysis on the miRNA discovered in the tumor sample and the adjacent healthy tissue sample (3:309) and draw the volcano plot. We selected |log2FC| > 1 and P.adj <0.05 as the screening criteria for significantly differentially expressed genes. Hsa‐mir‐133a‐2, which has a significantly reduced expression in cervical cancer, was selected for follow‐up research. We utilized the clinical information in TCGA‐CESC to perform Kaplan–Meier (K‐M) survival analysis on hsa‐mir‐133a‐2 in order to evaluate the prognostic value.

### 2.2 Prediction of hsa‐mir‐133a‐2 target location

The TargetScan database (http://www.targetscan.org/) is a commonly used miRNA target gene database. In mammals, miRNAs perform post‐transcriptional control by binding to the 3′Untranslated Region (3′UTR) inside the transcript sequence. The database integrates existing 3′UTR annotations and 3P‐seq sequencing results from the National Coalition Building Institute (NCBI) to provide a comprehensive transcript sequence for the corresponding 3′UTR region. We conducted research on human hsa‐mir‐133a‐2 in TargetScan database, and predicted target genes based on the conserved 8mer, 7mer, and 6mer locations that correspond to the miRNA seed region. The context score with a percentile >90 was selected as the screening criteria for hsa‐mir‐133a‐2 potential mRNA targets. In addition, we screened the considerably high expressed mRNA in cervical cancer tissue using the same way as described above for miRNA differential gene analysis. To further narrow the range of potential mRNA target genes, the intersection of mRNA target genes predicted by highly expressed mRNA and TargetScan database was selected.

Finally, K‐M survival analysis was performed on the above‐mentioned potential mRNA target genes and we also evaluated the influence of the expression of the above‐mentioned potential targets on progression‐free survival period(PFS)and overall survival (OS) of cervical cancer patients. We defined *p* < 0.05 as the point at which target expression is strongly linked to the survivability of patients diagnosed with cervical cancer.

### 2.3 KEGG enrichment analysis

LAMB3 function enrichment analysis is carried out using the “clusterProfiler” software. According to the median of LAMB3 expression, we divided all patients′ data within TCGA‐CESC project high expression groups and low expression groups. We utilized “edgeR” package of R software in order to normalize the expression matrix of the two sets of samples. Empirical Bayes Method was also employed to obtain the t value of the whole genome to assess the significance of the difference in gene expression. The standard of |log2FC| >1 and P.adj <0.05 was used to screen the significant differentially expressed genes between the two groups. In addition, the KEGG enrichment analysis was carried out. Pathways with a low FDR ‐ false discovery rate, of < 0.05 were considered to be significantly related to LAMB3.

### 2.4 Cell lines and cell culturation

We purchased two types of human cervical cancer cells named siha and caski from Shanghai Institute of Cell Biology Cell Bank (Chinese Academy of Sciences). We cultured human normal cervical cells(HUCEC) and caski in complete RPMI 1640 medium (GIBCO, USA) that containing 10% FBS and1% 100 U/ml penicillin/streptomycin. We cultivated siha cells in complete DMEM medium (GIBCO, USA) which contains 10% FBS and 1% 100 U/ml penicillin/streptomycin. All cells mentioned were cultured in an incubator with a temperature of 37°C and 5% CO2 level. Cells that required to be treated with phosphatidylinositol 3‐kinase (PI3K) inhibitor LY294002 (Cell Signaling Technology) were pre‐added with the inhibitor at a concentration of 10 μM and then incubated for 1 h.

### 2.5 Plasmid construction and cell transfection

hsa‐mir‐133a‐2 mimic and its corresponding negative control miRNA (miR‐mimic‐NC), hsa‐mir‐133a‐ 2 inhibitor and its corresponding negative control (miR‐inhibitor‐NC), LAMB3 overexpression vector (pcDNA‐LAMB3) as well as blank vector (pcDNA) were designed and synthesized by GenePharma (Shanghai, China). We studied and followed the Lipofectamine 2000 methods from Invitrogen (USA) and transfected siha and caski cells with 2 μg synthetic vector and 10 mM synthetic oligonucleotide. The transfected cells were routinely cultured for the subsequent experiments.

### 2.6 Extraction of RNA as well as real‐time fluorescent quantitative polymerase chain reaction (qRT‐PCR)

We extracted overall RNA from cervical cancer cells utilizing TRIzol reagent manufactured by Invitrogen (USA). We also reverse transcribed RNA into cDNA using Exiqon Universal cDNA Synthesis Kit II manufactured by Qiagen (Germany). We employed SYBR Green Real‐Time PCR Master Mixes manufactured by Qiagen (Germany) for qRT‐PCR experiments. In order to normalize hsa‐mir‐133a expression, we used U6 as the internal reference gene. We also utilized GAPDH as the internal reference gene in order to normalize LAMB3 expression. Hsa‐mir‐133a and LAMB3 relative expression level in cells were calculated by means of 2^−ΔΔCT^. All primers adopted during qRT‐PCR were synthesized by Shenggong Biotechnology (Table 1).

**TABLE 1:** Primers in the qRT‐PCR experiment

Genes	Primers	
GAPDH	F	5′‐TGCACCACCAACTGCTTAGC‐3′
	R	5′‐GGCATGGACTGTGGTCATGAG‐3′
U6	F	5′‐CTCGCTTCGGGCAGCACA‐3′
	R	5′‐CGCTTCACGAATTTGCGT‐3′
hsa‐mir‐133a	RT	5′‐CTCAACTGGTGTCGTGGAGTCGGCAATTCAGTTGAGCAGCTGGT‐3′
	GSF	5′‐CGGCGGTTTGGTCCCCTTCAAC‐3′
LAMB3	F	5′‐CCAAAGGTGCGACTGCAATG‐3′
	R	5′‐AGTTCTTGCCTTCGGTGTGG‐3′

### 2.7 Western blot experiment

We extracted total protein in cervical cancer cells using RIPA lysis buffer from Beyotimes (China), which contained 1% Phenylmethanesulfonyl fluoride (PMSF) and phosphatase inhibitor. Utilizing the bicinchoninic acid (BCA) method (Beyotimes, China), we determined the concentration level of extracted protein. The protein samples were diluted with phosphate buffered saline(PBS), then 5 × loading buffer was added. We heated samples at 99°C for 15 min to denature the protein. After that, a 20 μg protein sample was added to each lane. We separated the total protein utilizing 10% SDS‐PAGE ‐ sodium dodecyl sulfate‐polyacrylamide gel electrophoresis. The samples were then transferred to a polyvinylidene fluoride (PVDF) membrane. After blocking samples with skimmed milk powder with a concentration level of 5% at room temperature for 1 h, we placed PVDF in a specific primary antibody. We then incubated them at 4°C overnight. The membrane was then treated with a secondary antibody at room temperature once more. After 1 h, the enhanced chemiluminescence detection kit (Beyotimes, China) was used to identify the protein lanes, and the gray value of each lane was calculated by Image J software. We treated glyceraldehyde‐3‐phosphate dehydrogenase(GAPDH) as an internal reference. The primary antibodies involved in this research include LAMB3 1:1000 dilution (Proteintech, China), GAPDH 1:1000 dilution (Proteintech, China), p‐AKT 1:1000 dilution (Proteintech, China), p‐PI3K 1:1000 dilution (Bioss, China), AKT 1:1000 dilution (Proteintech, China), PI3K 1:1000 dilution (Proteintech, China). We adopted HRP ‐ horseradish peroxidase as secondary antibodies ‐conjugated rabbit antibody with 1:5000 dilution (Proteintech, China).

### 2.8 Cell proliferation and invasion

Firstly, the proliferation ability of cervical cancer cells was determined by cell counting kit‐8(CCK8) trial. The transfected cells were then seeded in a 96‐well plate for 24 to 72 h. Those wells have a density of 5 × 10^3^ cells per well. At 24, 48, and 72 h after inoculation, the original medium in each well was removed and complete 100 μl fresh complete medium, as well as 10 μl CCK8 reagent manufactured by Beyotimes (China), were added. After incubating those samples for 2 h in an incubator, we adopted a microplate reader to determine the absorbance of each well at 450 nm.

Then, the invasion ability of cervical cancer cells after transfection was determined using transwell method. First, we prepared Matrigel gel (BD Biosciences) utilizing a serum‐free medium and applied it to the upper chamber of the transwell. We also adopted a serum‐free medium in order to prepare the cell suspension of 5 × 10^4^ cells/m and inoculated it in the upper chamber coated with Matrigel. We then added 600 μl medium containing 10% FBS to the lower chamber. 48 h after normal incubation, we utilized 4% paraformaldehyde and fixed migrated cells located on the lower surface of the membrane. We then decided to stained those cells with crystal violet dye (0.1%) after 30 min. The period of the dying process was set to 10 min. Finally, we selected 5 fields of view from each group of cells at random to observe the cells in the lower chamber under the microscope. We took pictures and counted numbers.

### 2.9 Luciferase reporter gene detection

We amplified the wild‐type or mutant(WT or MUT) LAMB3 3′‐UTR segments that carry potential binding site of hsa‐mir‐133a‐2 and insert them into the pGL3 vector to prepare LAMB3‐WT and LAMB3‐MUT Luciferase reporter plasmid. Then, utilizing Lipofectamine 2000 (Invitrogen, USA), 40 nM hsa‐mir‐133a‐2 mimic, miR‐NC or hsa‐mir‐133a‐2 inhibitor was co‐transformed into siha and caski cells with 100 ng pGL3‐3′UTR‐WT/MUT. Each cell was prepared into the following 5 cell groups: LAMB3‐WT + NC, LAMB3‐WT + hsa‐mir‐133a‐2, LAMB3‐WT + hsa‐mir‐133a‐2 + inhibitor, LAMB3‐Mut + NC and LAMB3‐Mut + hsa‐mir‐133a‐2. 48 h right after transfection, we employed a dual luciferase assay system to evaluate the luciferase activity of each cell group.

### 2.10 Statistical analysis

All experimental data are acquired from 3 independent repeating experiments. Experimental data are expressed as mean ± standard deviation (SD). We then conducted statistical analysis utilizing GraphPad Prism 8 software. We used Student's *t*‐test (two‐tailed, paired, applied such test to two independent samples) or one‐way analysis of variance (ANOVA) combined with Dunnett's post hoc tests (applied to three sets of samples and above independent samples) to perform statistical analysis on each group. When *p*‐value <0.05, we considered the differences to be statistically significant.

## 3 RESULTS

### 3.1 hsa‐mir‐133a‐2 expression is significantly low in cervical cancer, which has a protective effect on the prognosis

**FIGURE 1:**
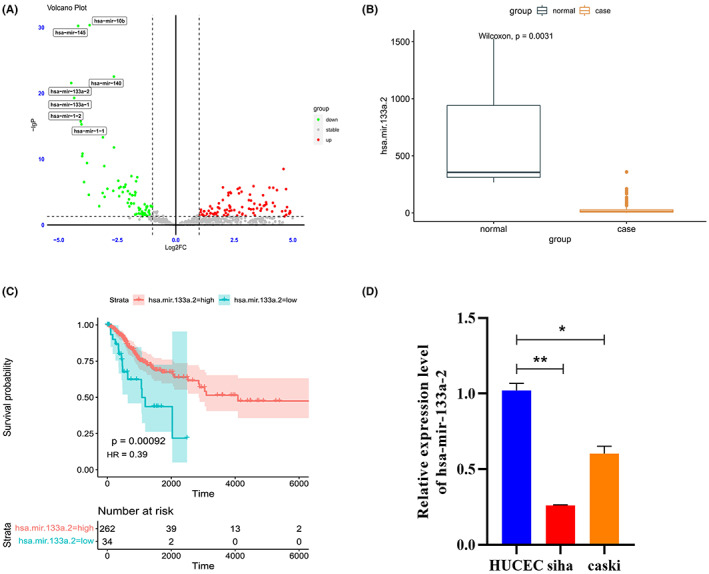
Hsa‐mir‐133a‐2 expression was discovered in samples and cells of cervical cancer patients. (A) Screening out significantly low‐expressed miRNAs in cervical cancer using differential gene analysis on the TCGA‐CESCmiRNA expression profile. (B) Compare differential expression of hsa‐mir‐133a‐2 in 309 tumor samples and 3 normal tissue samples in TCGA‐CESC utilizing the Wilconxon rank sum test. (C) Explore the prognostic significance of hsa‐mir‐133a‐2 within cervical cancer by performing K‐M survival analysis on 307 cervical cancer patients identified with high/low expression of hsa‐mir‐133a‐2 miRNA in TCGA‐CESC. (D) The hsa‐mir‐133a‐2 expression in human healthy cervical cells and cervical cancer cells was detected by utilizing qRT‐PCR, *p < 0.05, **p < 0.01.

TCGA‐CESC miRNA difference analysis results demonstrated that 54 miRNAs are significantly low expressed in clinical samples of cervical cancer (Figure 1A). Among them, mir‐10b, mir‐145 and mir‐140 have all been studied to be related to cervical cancer. Therefore, the purpose of this study is to find out the extensive function that hsa‐mir‐133a‐2 plays in the progression of cervical cancer.

We can find in Figure 1B that hsa‐mir‐133a‐2 expression was considerably lower in 309 tumor samples from TCGA‐CESC than its expression in 3 samples of adjacent healthy tissues (Figure 1B). Later, the prognostic value of hsa‐mir‐133a‐2 has in cervical cancer prognosis was evaluated utilizing K‐M survival analysis. The analytical results demonstrated that patients with high level of expression of hsa‐mir‐133a‐2 have a relatively long overall survival period. That is, hsa‐mir‐133a‐2 demonstrated a protective effect on cervical cancer prognosis (Figure 1C). The baseline data of the patients studied in our experiments are shown in Table 2.

**TABLE 2:** Baseline data of patients included in the survival analysis

Clinicopathological factors	Number of patients (person)	Proportion (%)
Age		
<60	241	78.50%
≥60	66	21.50%
Gender		
Male	0	0
Female	307	100%
Survival state		
Alive	236	76.87%
Deceased	71	23.13%

In order to investigate the difference in hsa‐mir‐133a‐2 expression level found in normal and tumor tissues, our study analyze hsa‐mir‐133a‐2 expression in HUCECand human cervical cancer cells named siha and caski by qRT‐PCR technology. The results demonstrated that compared to normal cervical cells, hsa‐mir‐133a‐2 was significantly down‐regulated in siha and caski, the two cervical cancer cell lines (Figure 1D, *p* < 0.05).

### 3.2 hsa‐mir‐133a‐2 inhibits cervical cancer cell proliferation and invasion in vitro

**FIGURE 2:**
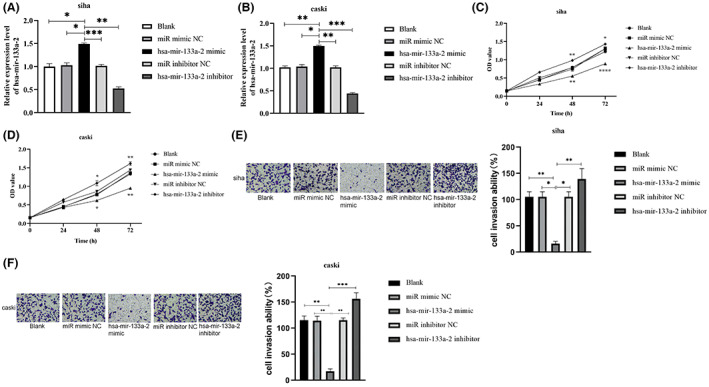
The effect of hsa‐mir‐133a‐2 has on cervical cancer cell proliferation and invasion in vitro. (A, B) Transfecting siha/caski cells respectively with hsa‐mir‐133a‐2 mimic, hsa‐mir‐133a‐2 inhibitor, miR‐mimic‐NC and miR‐inhibitor‐NC in order to regulate hsa‐mir‐133a‐ 2 expression level while verifying transfection utilizing qRT‐PCR experiment. (C, D) Determine the effect of expression of hsa‐mir‐133a‐2 on cervical cancer cell proliferation utilizing CCK8 trial. (E, F) Investigate the effect of hsa‐mir‐133a‐2 expression has on the invasion of cervical cancer cells using transwell invasion experiment, *p < 0.05, **p < 0.01, ***p < 0.001.

Our study carried out CCK8 experiments and transwell invasion test to verify the role that hsa‐mir‐133a‐2 plays on cervical cancer cell proliferation and invasion in vitro at cellular level. Cell transfection was adopted in order to regulate hsa‐mir‐133a‐2 expression in cervical cancer cells. We used hsa‐mir‐133a‐2 mimic, miR‐mimic‐NC, hsa‐mir‐133a‐2 inhibitor, as well as miR‐inhibitor‐NC transfect into siha and caski, and hsa‐mir‐133a‐2 expression in the transfected cells was verified by the qRT‐PCR trial. Figure 2A,B shows that hsa‐mir‐133a‐2 expression was much greater in hsa‐mir‐133a‐2 mimic transfected cells than in the blank group or the comparable negative control group (miR mimic NC). Moreover, the expression of hsa‐mir‐133a‐2 was significantly increased in the cervical cancer cells transfected with hsa‐mir‐133a‐2 mimic (*p* < 0.05), while significantly decreased in the inhibitor group (*p* < 0.05), indicating that the transfection was successful. There was no significant difference in hsa‐mir‐133a‐2 in the mimic/inhibitor NC group and the blank group.

CCK8 and transwell invasion experiments were performed on the transfected cells aimed to assess the role of hsa‐mir‐133a‐2 knockdown in cervical cancer cell proliferation and invasion. The experimental results are shown in Figure 2C–F. The simulated treatment with hsa‐mir‐133a‐2 decreased viability and invasion ability of siha and caski cells. When siha and caski cells were treated with a hsa‐mir‐133a‐2 inhibitor, their vitality and invasion capacity increased.

To sum up, our findings suggested that hsa‐mir‐133a‐2 has inhibitory effects on cervical cancer cell proliferation and invasion.

### 3.3 Downstream target gene of hsa‐mir‐133a‐2 ‐ LAMB3, hsa‐mir‐133a‐2 negatively regulates LAMB3 expression level

**FIGURE 3:**
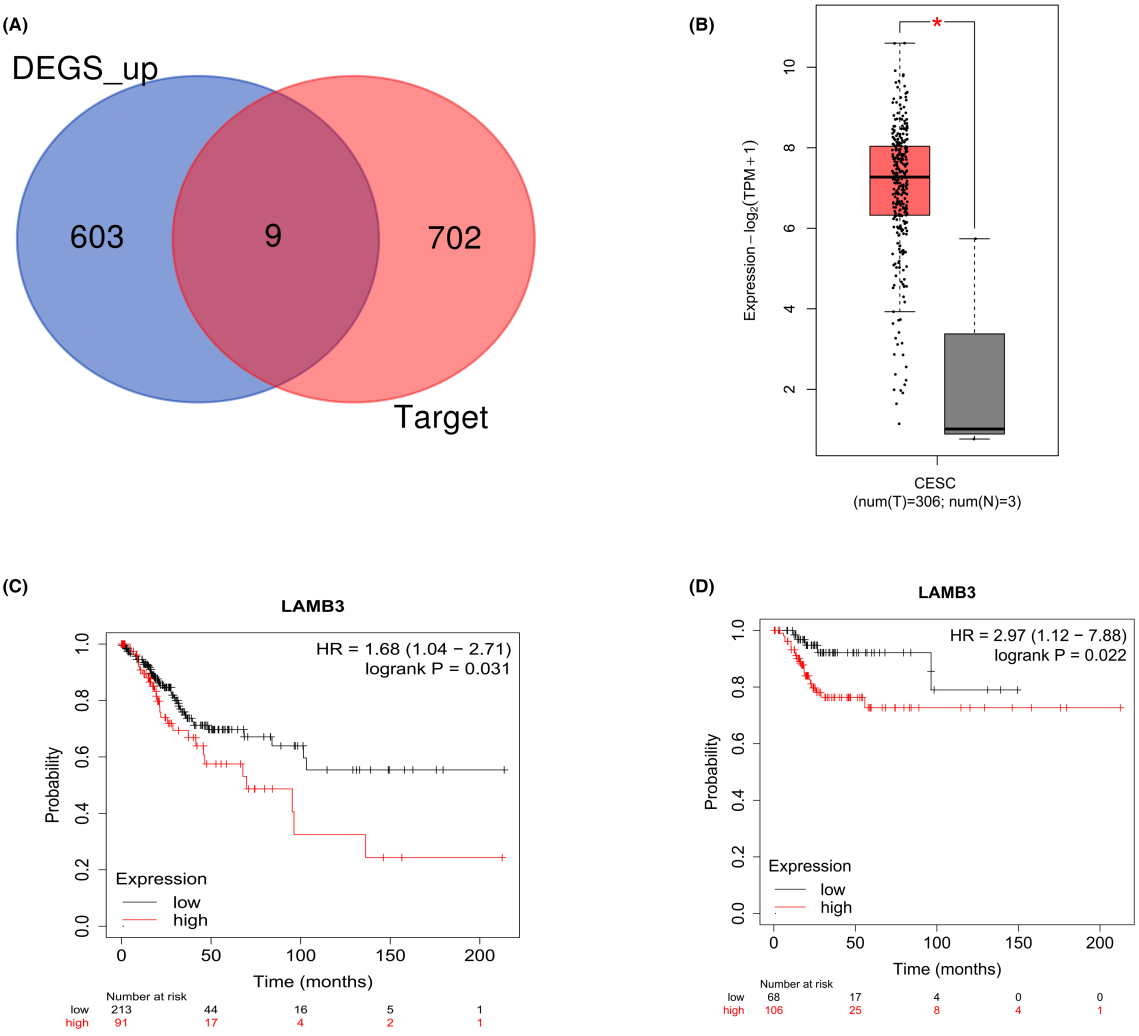
Screening and determination of downstream target genes of hsa‐mir‐133a‐2. (A) Obtain significantly high expression mRNA through TCGA‐CESC mRNA difference analysis, and cross the result with hsa‐mir‐133a‐2 predicted target gene from TargetScan database, and screen preliminarily the potential hsa‐mir‐133a‐2 target genes. (B) LAMB3 expression in TCGA‐CESC tumor tissue and normal tissues (*p < 0.05). (C, D) The K‐M survival analysis of 307 cervical cancer patients with high and low hsa‐mir‐133a‐2 expressions found in TCGA‐CESC was performed in order to explore the relationship between hsa‐mir‐133a‐2 expression and the OS (C) as well as PFS (D) of cervical cancer patients.

In order to determine hsa‐mir‐133a‐2 downstream target genes in cervical cancer, our study adopted a combination of mRNA difference analysis and TargetScan database prediction to conduct a preliminary screening of potential target genes. First, the differential analysis of TCGA‐CESC mRNA expression data was performed, and 612 mRNAs with significantly up‐regulated expression were obtained (|log2FC| > 1, *p* < 0.05). Secondly, we predicted hsa‐mir‐133a‐2 target genes by utilizing TargetScan database. A total of 711 predicted mRNA target genes were acquired. We discovered 9 mRNAs that are likely to be downstream target genes of hsa‐mir‐133a‐2 by intersecting and cross‐comparing 711 predicted mRNAs with mRNAs that showed significant‐high expression in the aforementioned cervical cancer, namely FOXQ1, PTPRZ1, ELF3, CTSV, ARNTL2, LAMB3, RAB27B, E2F7, and CDCA8 (Figure 3A).

Then, the association between the nine mRNAs mentioned above and the survival states of cervical cancer patients was next investigated by using K‐M survival analysis. The results indicate that only the *p* values of PFS and OS of LAMB3 are < 0.05. As a result, LAMB3 is selected as the target gene for further research. As shown in Figure 3B, LAMB3 expression in cervical cancer tissues is noticeably higher than in adjacent paracancerous tissues. Figure 3C,D demonstrated that LAMB3 expression is significantly correlated with the PFS and OS of cervical cancer patients negatively. That is, the higher the level of LAMB3 expression, the shorter the progression‐free survival period (PFS)and OS of cervical cancer patients.

**FIGURE 4:**
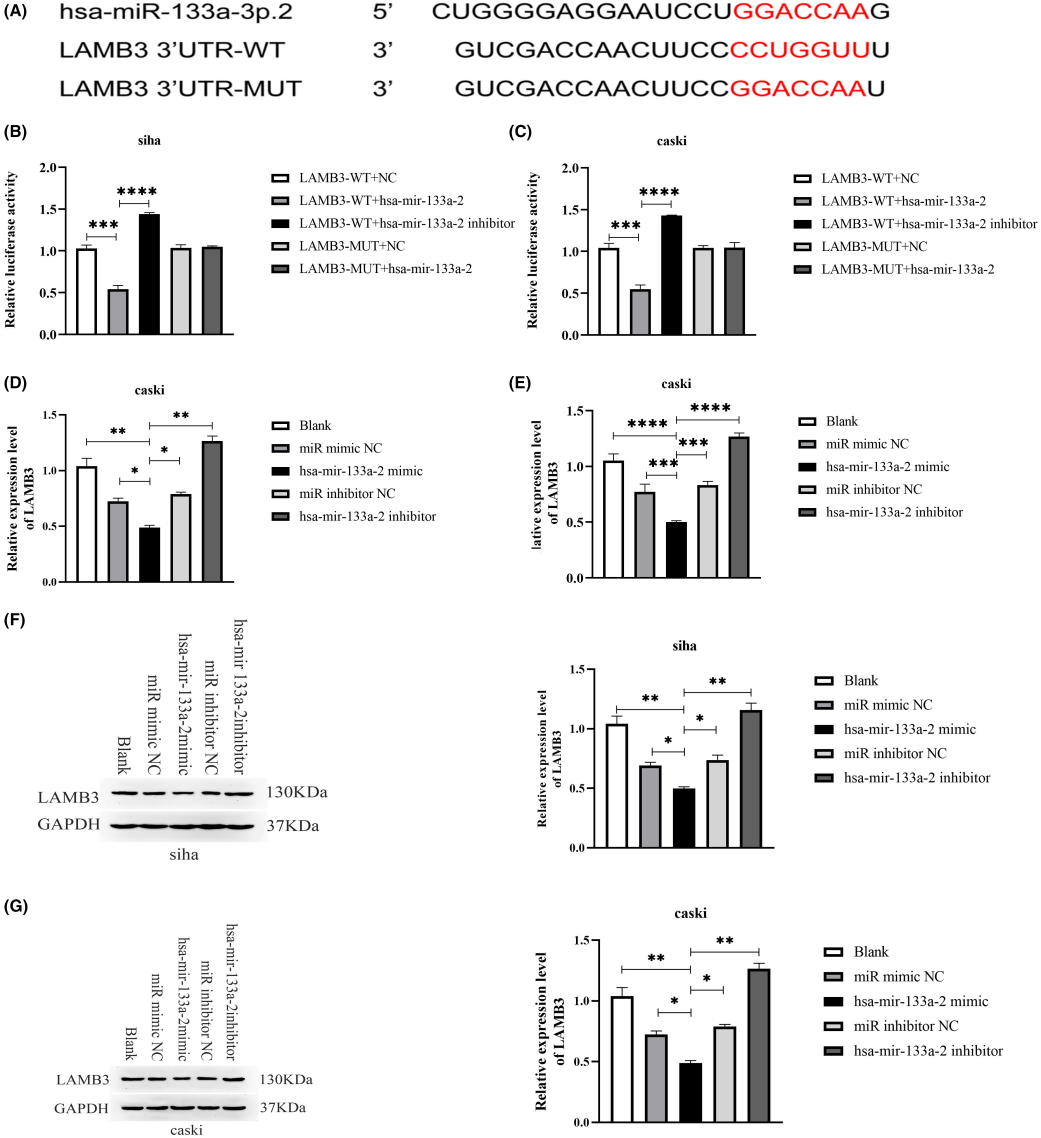
Determine the interaction between LAMB3 3′ ‐UTR and hsa‐mir‐133a‐2 in cervical cancer cells. (A) Potential binding sites of LAMB3 3’‐UTR and hsa‐mir‐133a‐2. (B, C) Interactions between hsa‐mir‐133a‐2 and potential binding locations in LAMB3 3′ ‐UTR was researched by using luciferase reporter gene detection. The results demonstrated that hsa‐mir‐133a‐2 has a significant inhibitory effect on the luciferin of wild‐type LAMB3 3′ ‐UTR enzyme activity but has almost no effect on mutant LAMB3 3′ ‐UTR luciferase activity. It proved that hsa‐mir‐133a‐2 directly targets LAMB3. (D, E) Utilizing qRT‐PCR to anaylize LAMB3 expression in siha and “caskiz” cells that were transfected with hsa‐mir‐133a‐2 inhibitor NC, hsa‐mir‐133a‐2 mimic as well as miR mimic NC, hsa‐mir‐133a‐2 inhibitor to verify the direct regulation of LAMB3 expression in cervical cancer cells. (F, G) LAMB3 expression in siha and caski cells that were transfected with hsa‐mir‐133a‐2 inhibitor NC, hsa‐mir‐133a‐2 mimic, miR mimic NC and hsa‐mir‐133a‐2 inhibitor was analyzed by Western blotting in order to examine direct regulatory ability of hsa‐mir‐133a‐2 has on LAMB3 expression in cervical cancer cells, *p < 0.05, **p < 0.01, ***p < 0.001.

The TargetScan database prediction findings revealed that LAMB3 and hsa‐mir‐133a‐2 share a common location. The location is found at 309–315 bp in the LAMB3 3’‐UTR sequence (Figure 4A). We predicted it to be hsa‐mir‐133a −2 binding site.

We adopted siha and caski cells for luciferase reporter gene detection. Our study results demonstrated that the luciferase activity of cervical cancer cells co‐transfected with LAMB3‐WT and hsa‐mir‐133a‐2 was significantly reduced compared to the NC control group. The luciferase activity of the cervical cancer cells that co‐transfected with LAMB3‐WT as well as hsa‐mir‐133a‐2 inhibitor was increased greatly. The findings of the experiments showed that hsa‐mir‐133a‐2 overexpression greatly inhibited the luciferase activity of the wild‐type LAMB3 3′UTR. However, the luciferase activity of mutant LAMB3 3′UTR's was relatively unchanged compared to the NC control group. As a result, when the binding site on LAMB3 is mutated, hsa‐mir‐133a‐2 could not bind to the LAMB3 binding site, and the luciferase activity is no longer inhibited. The above results demonstrated that hsa‐mir‐133a‐2 negatively regulates the expression of LAMB3 by connecting to the predicted LAMB3 3′‐UTR binding site depicted in Figure 4B,C.

To better confirm the regulatory effect of hsa‐mir‐133a‐2 has on LAMB3 expression, siha and caski cells were transfected with hsa‐mir‐133a‐2 simulation, miR simulation NC, hsa‐mir‐133a‐2 inhibitor, and hsa‐mir‐133a‐2 inhibitor NC. The RT‐PCR results showed LAMB3 expression level decreased in cells that overexpress hsa‐mir‐133a‐2, while LAMB3 expression level recovered in cells treated with hsa‐mir‐133a‐2 inhibitor (Figure 4D,E). WB results also confirmed that hsa‐mir‐133a‐2 expression significantly inhibited LAMB3 expression at the protein level. (Figure 4F,G). In summary, the results demonstrated that hsa‐mir‐133a‐2 could directly bind to LAMB3 and inhibited its expression.

### 3.4 LAMB3 could reverse the inhibitory effect of hsa‐mir‐133a‐2 on tumors

LAMB3 expression levels and hsa‐mir‐133a‐2 levels in cervical cancer cells were adjusted to evaluate whether LAMB3 has an effect on hsa‐mir‐133a‐2's role in cervical cancer cells. The cervical cancer cell proliferation and invasion tables were examined as well. We co‐transfected siha and caski cells with hsa‐mir‐133a‐2 with LAMB3 overexpression vector (pcDNA‐LAMB3) or blank vector (pcDNA). Moreover, hsa‐mir‐133a‐2 mimic and its corresponding negative control ‐ miR‐mimic‐NC, were also transfected into siha and caski cells.

**FIGURE 5:**
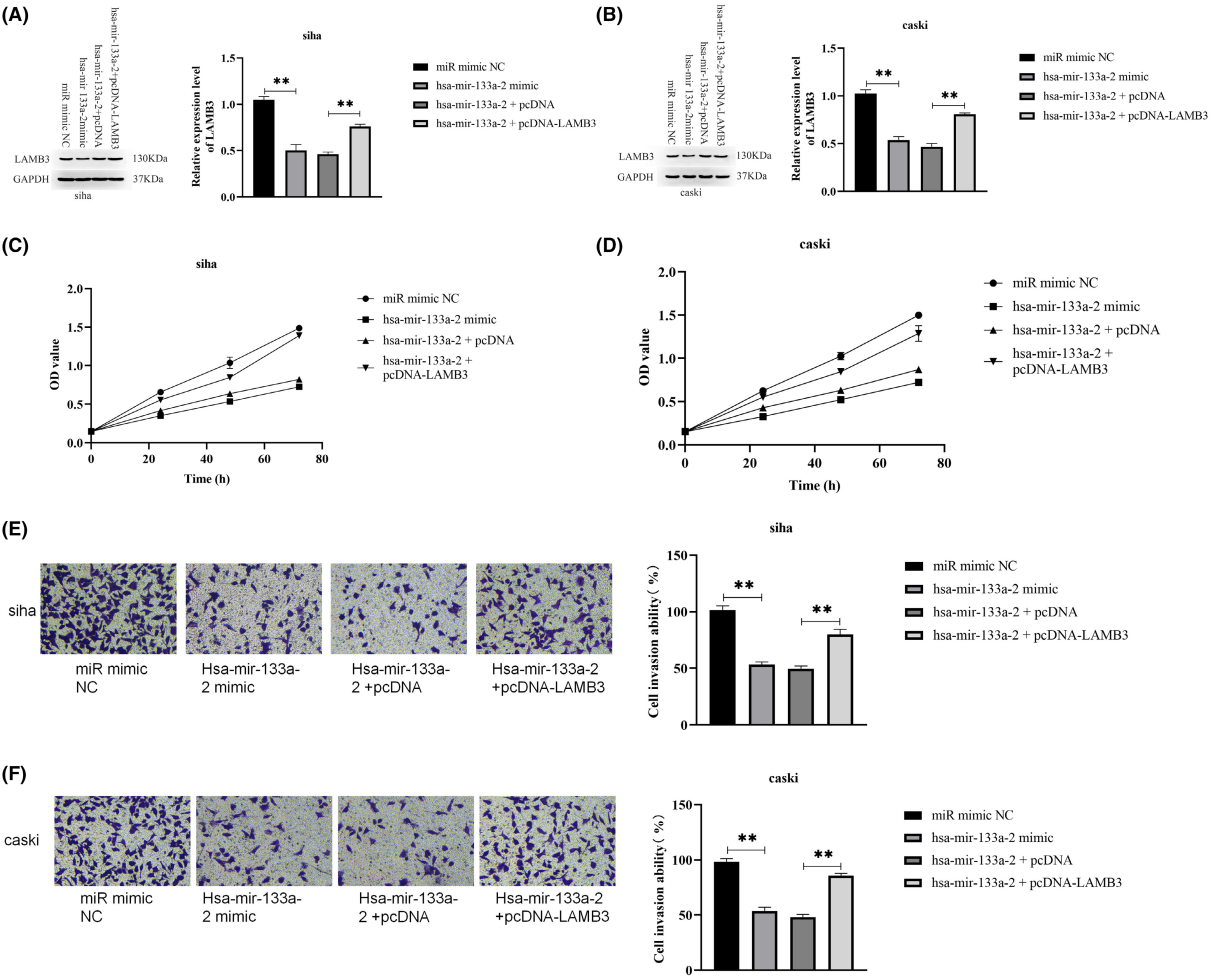
Verification of LAMB3's ability in reversing tumor‐suppressive effect of hsa‐mir‐133a‐2 has in cervical cancer. (A, B) Examine LAMB3 expression level in transfected cells utilizing WB experiment to verify the regulation of hsa‐mir‐133a‐2 on LAMB3 expression in cervical cancer cells and whether our transfection experiment is successful. (C, D) The CCK‐8 experiment verified that the up‐regulation of LAMB3 reversed hsa‐mir‐133a‐2 inhibitory effect on cervical cancer cell proliferation. (E, F) Through the transwell invasion experiment, we verified that LAMB3 overexpression reversed hsa‐mir‐133a‐2 inhibitory effect of on the invasion of cervical cancer cells, *p < 0.05, **p < 0.01, ***p < 0.001.

In our study, WB experiment was adopted in order to detect LAMB3 expression level in the transfected cells. Our experimental results demonstrated that the overexpression of hsa‐mir‐133a‐2 inhibits the expression level of LAMB3, while the upregulation of LAMB3 could reverse the inhibition caused by the hsa‐mir‐133a‐2 mimic in siha and caski cells (*p* < 0.05) (Figure 5A). The finding indicated that our transfection trial was successful.

The transfected cells mentioned above were utilized for CCK‐8 trial and transwell invasion experiment respectively. The overexpression of hsa‐mir‐133a‐2 inhibited the proliferation of siha and caski cells, according to CCK‐8 results, however, the upregulation of LAMB3 expression significantly decreased the inhibitory effect of hsa‐mir‐133a‐2 on cervical cancer cell proliferation (Figure 5B, *p* < 0.05). The results of transwell invasion experiments demonstrated that the overexpression of hsa‐mir‐133a‐2 has a significant inhibitory effect on the cell invasion of siha and caski, while the promotion of LAMB3 expression level weakens the ability of hsa‐mir‐133a‐2 to inhibit the invasion of cervical cancer cells, *p* < 0.05 (Figure 5C).

Above mentioned experimental results indicated that LAMB3 high expression could reverse the inhibition of cervical cancer cell proliferation and invasion caused by hsa‐mir‐133a‐2 expression. hsa‐mir‐133a‐2 inhibits cervical cancer cell proliferation and invasion by targeting LAMB3.

### 3.5 hsa‐mir‐133a‐2 inhibits PI3K/AKT signaling in cervical cancer cells by targeting LAMB3

**FIGURE 6:**
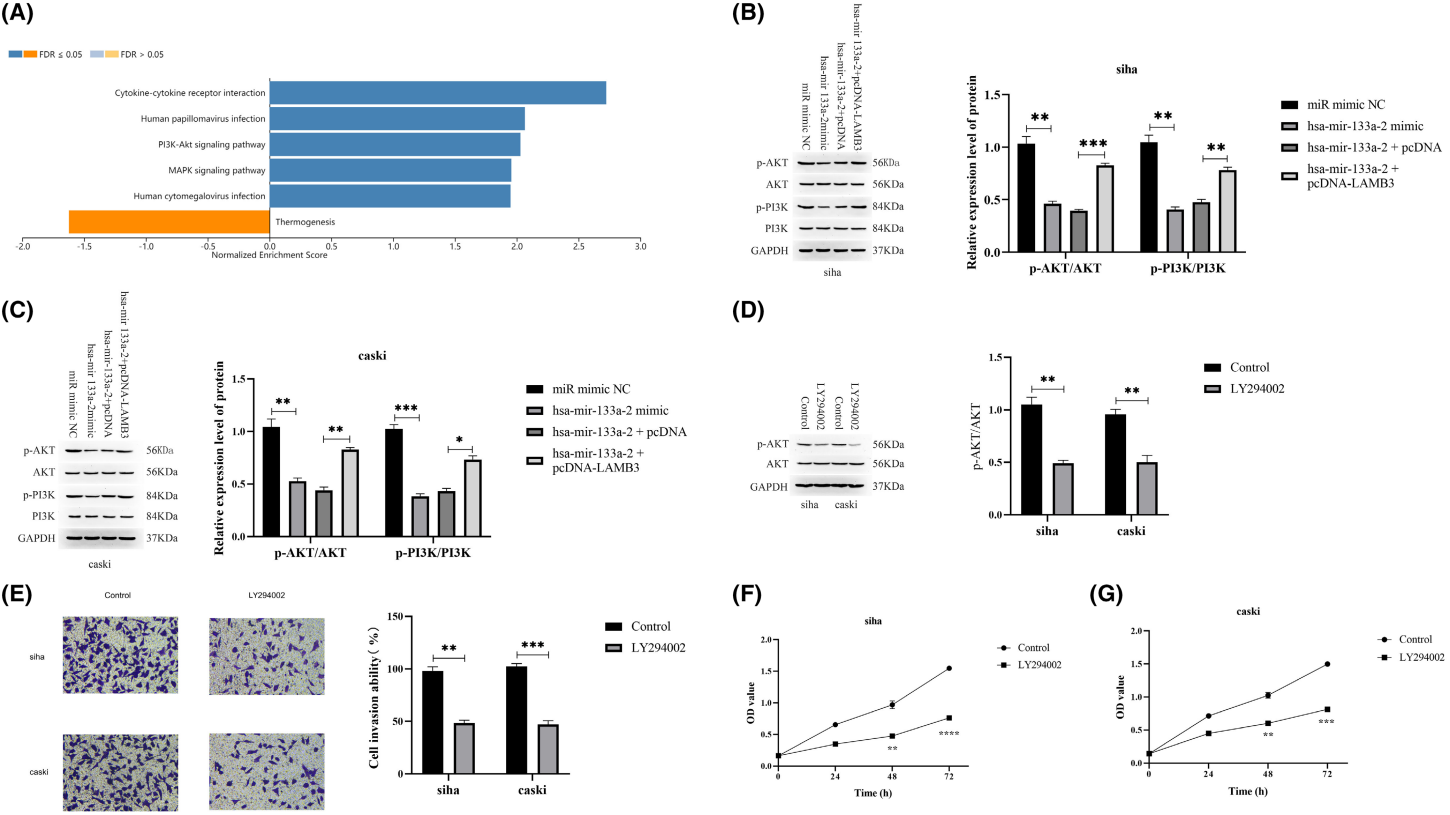
The regulatory role of hsa‐mir‐133a‐2 in PI3K/AKT signaling pathway in cervical cancer cells by targeting LAMB3. (A) To perform pathway enrichment analysis (KEGG) on LAMB3 differential genes high/low expression group to study the functional pathways closely related to LAMB3. (B, C) Investigate hsa‐mir‐133a‐2 and LAMB3 expression regulations in cervical cancer cells on signaling pathway PI3K/AKT through WB experiments. (D) Utilize AKT inhibitor LY294002 to regulate the phosphorylation level of AKT in siha and caski cells. (E) Analyze phosphorylated AKT in cervical cancer cell invasion by using transwell invasion experiment. (F, G) The effect of phosphorylated AKT on cervical cancer cell growth was detected using the CCK8 assay. *p < 0.05, **p < 0.01, ***p < 0.001.

We selected to explore the underlying mechanism of LAMB3's role in the proliferation and invasion of cervical cancer cells, and our study performed KEGG pathway enrichment analysis on the differential genes within high/low expression LAMB3 group. The results demonstrated that the LAMB3 gene is related to the PI3K/ATK pathway (Figure 6A,B). It is speculated that hsa‐mir‐133a‐2 may regulate this pathway by targeting LAMB3.

The results of WB experiments demonstrated that overexpression of hsa‐mir‐133a‐2 dramatically reduced phosphorylation levels of AKT and PI3K proteins; however, phosphorylation levels recovered with the up‐regulation of LAMB3. We also found out that protein levels of AKT and PI3K in siha and caski cells were relatively stable in each cell group (Figure 6B,C). It indicated that hsa‐mir‐133a‐2 indirectly regulates the signaling pathway of PI3K/AKT by inhibiting LAMB3.

**FIGURE 7:**
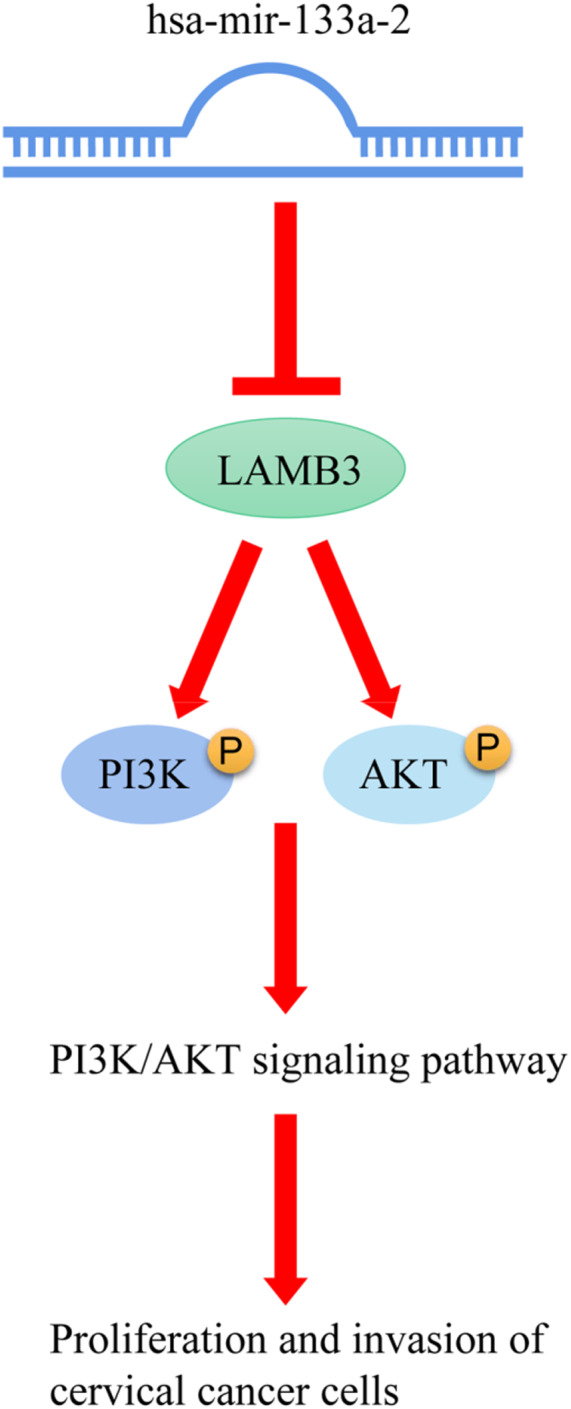
This study found that hsa‐mir‐133a‐2 directly targets LAMB3 and negatively regulates its expression in cervical cancer. Hsa‐mir‐133a‐2 inhibits PI3K/AKT pathway by targeting LAMB3, hence inhibiting the proliferation and invasion of cervical cancer cells.

By targeting LAMB3, the above investigations demonstrated that hsa‐mir‐133a‐2 could inhibit proliferation and invasion of cervical cancer cells, as well as the activation of the PI3K/AKT signaling pathway. However, it is unknown whether hsa‐mir‐133a‐2 could inhibit proliferation and invasion of cervical cancer cells by regulating pathway of PI3K/AKT. In order to further verify PI3K/AKT pathway function in cervical cancer cells, the AKT inhibitor LY294002 was adopted to inhibit the phosphorylation level of AKT existing in siha and caski cells, as demonstrated in Figure 6D. Figure 6E,F show that cervical cancer cell proliferation and invasion ability in the LY294002 treatment group was significantly reduced compared to the related control group, indicating that the decrease in AKT expression level has a significant inhibitory effect on cervical cancer cell proliferation and invasion. In summary, hsa‐mir‐133a‐2 could inhibit the pathway of PI3K/AKT by targeting LAMB3 in cervical cancer, thereby inhibiting cervical cancer cell proliferation and invasion (Figure 7).

## 4 DISCUSSION

Our study discovered that the expression of hsa‐mir‐133a‐2 decreased in cervical cancer. And the expression of hsa‐mir‐133a‐2 is positively correlated to the overall survival period of cervical cancer patients. As a result, it is speculated that this miRNA may play an important role in the development of cervical cancer. Therefore, our study focuses on the effects of hsa‐mir‐133a‐2 on cervical cancer cell proliferation and invasion as well as the underlying molecular mechanisms. The findings of the experiments demonstrated that hsa‐mir‐133a‐2 has a significant inhibiting effect on cervical cancer cell proliferation and invasion. Furthermore, our study is the first to use a combination method of TargetScan database prediction, differential mRNA analysis, and KM survival analysis to search the downstream mRNA target genes of hsa‐mir‐133a‐2. Our study concluded LAMB3 as a potential target gene associated with the survival of cervical cancer patients. LAMB3 has been identified as a direct target of hsa‐mir‐133a‐2 by mechanism studies. Meanwhile, there is a negative correlation between the expression levels of hsa‐mir‐133a‐2 and LAMB2, and LAMB3 could reverse hsa‐mir‐133a‐2 inhibitory effect on cervical cancer cell proliferation and invasion. It was clear from further functional enrichment analysis findings that LAMB3 is connected to the PI3K/AKT signaling pathway. Based on such results, it is speculated that hsa‐mir‐133a‐2 may regulate the pathway by targeting LAMB3, thus affecting cervical cancer cell proliferation and invasion. As a result, hsa‐mir‐133a‐2 may be a promising novel therapeutic target for cervical cancer molecular targeted therapy. Inhibiting hsa‐mir‐133a‐2 expression in patients may prevent the further development of cervical cancer.

MiRNAs have gotten a lot of attention recently as a novel type of therapeutic target in tumor molecular targeted therapy. In mammals, miRNA mainly binds to the 3′UTR, of mRNA to guide target genes into the “5′ to 3′mRNA degradation pathway” thus inhibiting post‐transcriptional translation of mRNA.^18^ As a result, the regulation of multiple biological processes and multiple pathways in tumors were altered. The genetic heterogeneity between tumor and normal tissues is the foundation of molecular targeted therapy. Based on the differences in the expression of miRNAs between various tissues, molecular targeted drugs can deliver drugs more effectively and specifically to the areas that require treatment. Such high treatment specificity greatly decreases therapeutic drug harm to normal tissues and has fewer side effects than conventional chemotherapy.^19^ In addition, miRNAs can also interact with small molecules such as extracellular vesicles (EVs), circularRNAs(circRNAs), and long non‐coding RNAs(lncRNAs) to jointly regulate tumor progression.^20^, ^21^, ^22^ Therefore, an in‐depth study of abnormally expressed miRNAs in tumors and evaluation of their value in molecular targeted therapy will be beneficial in promoting the development of tumor therapy.

Through the differential miRNA analysis of TCGA‐CESC and qRT‐PCR experiment at the cell level, our study discovered and verified that hsa‐mir‐133a‐2 expression level existing in cervical cancer was remarkably lower than that of adjacent noncancerous tissues and normal cervical cells. Increasing the expression of hsa‐mir‐133a‐2 in cervical cancer cells can inhibit cell proliferation and invasion. To some extent, knocking down hsa‐mir‐133a‐2 in cervical cancer cells might promote cell proliferation and invasion. The above results demonstrated that hsa‐mir‐133a‐2 is a type of cervical cancer tumor suppressor gene. Meanwhile hsa‐mir‐133a‐2 miRNA is also a member of the family member of miR‐133a. Numbers of previous studies demonstrated that miR‐133a is down‐regulated in various types of tumors and acts as a tumor suppressor in tumors. For example, Zhang et al. found that up‐regulation of microRNA‐133a in human glioma can inhibit tumor cell proliferation, migration and invasion through the JAK/STAT signaling pathway.^23^ MiR‐133a inhibits lung cancer cell proliferation in vivo and in vitro by targeting the LASP1 and TGF‐β/Smad3 signaling pathways.^24^ Research made by Tang et al. suggested that miR‐133a‐3p can block PI3K/AKT signaling by targeting MET, EGFR, IGF1R and FGFR1 and other cytokine receptors in pancreatic cancer to inhibit bone metastasis of pancreatic cancer.^25^ Furthermore, miR‐133a‐3p can regulate the PI3K/AKT/mTOR signaling pathway in thyroid cancer by modulating ZEB1‐AS1 and targeting LPAR3 and EGFR, inhibiting thyroid cancer cell proliferation while promoting cell death.^26^ Our study researched the molecular mechanism of hsa‐mir‐133a‐2 in cervical cancer cell proliferation and invasion. We then discovered that hsa‐mir‐133a‐2 targets LAMB3 and regulates the expression of LAMB3 negatively. By blocking the PI3K/AKT signaling pathway mediated by LAMB3, hsa‐mir‐133a‐2 can inhibit cervical cancer cell proliferation and invasion. Therefore, targeting hsa‐mir‐133a‐2 has great therapeutic potential for cervical cancer. Many miRNAs, in addition to hsa‐mir‐133a‐2, have been found to be abnormally appeared in cervical cancer. Those miRNAs could have a role in cervical cancer development by regulating the proliferation, metastasis, apoptosis, DNA repair, and other key biological processes of cervical cancer cells. These miRNAs have also been linked to clinical risk factors for cervical cancer.^27^, ^28^, ^29^ MiR‐125a‐5p, for example, inhibits cervical cancer cell proliferation both in vivo and in vitro by targeting GALNT7 to inhibit the signaling cascade EGFR/PI3K/AKT after significantly down‐regulating such miRNA in cervical cancer cells.^30^

LAMB3 is one of the three subunits involved in the coding of Laminin‐332(LM‐332) (the remaining two are LAMA3 and LAMC2) and is responsible for the coding of the β chain in LM‐332.^14^ LM‐332 is an extracellular matrix protein secreted by human keratinocytes. It is an important biologically active component in the basal laminar. It is vital for cell differentiation, migration, and adhesion, and cell proliferation and survival, and it has been linked to the progression of a variety of malignancies.^31^, ^32^, ^33^ A vast number of studies have shown that LAMB3 promotes the invasion and metastasis of various tumors such as thyroid cancer,^34^ colorectal cancer,^35^, ^36^ colon cancer,^37^ and pancreatic cancer^38^ by regulating pathways such as focal adhesions. Zhou et al. researched the role that SNPs ‐ single nucleotide polymorphisms, played in LAMB3 existing in cervical cancer and discovered that pri‐miR‐218 rs11134527 down‐regulated miR‐218 expression in cervical cancer, while LAMB3 rs2566 up‐regulated LAMB3 in cervical cancer. miR‐218′s direct target is LAMB3, and the changes in the expression of the two could promote the infection of the HPV virus to the surrounding tissues of the lesion, resulting in cervical cancer.^39^ Yamamoto et al. further demonstrated that miRNA‐218 inhibits cancer cell migration and invasion in cervical squamous cell carcinoma by targeting the focal adhesion pathway.^17^ Our experimental results indicated that overexpression of LAMB3 can reverse the inhibition of cervical cancer cell proliferation and invasion induced by hsa‐mir‐133a‐2. The results of survival analysis demonstrated that LAMB3 is correlated with the survivability of patients diagnosed with cervical cancer negatively, and acts as a carcinogen in cervical cancer.

The functional enrichment analysis of differential genes existed in LAMB3 high/low expression groups discovered that LAMB3 is closely linked to the PI3K/AKT signaling pathway, and the overexpression of hsa‐mir‐133a‐2 has a significant inhibitory effect on the protein phosphorylation level of AKT and PI3K. Increasing LAMB3 expression reversed the inhibitory effect of hsa‐mir‐133a‐2 on p‐AKT and p‐PI3K, which indicated that hsa‐mir‐133a‐2 can inhibit the activation of signaling pathway PI3K/AKT by means of targeting LAMB3. It is widely acknowledged that the PI3K/AKT signaling pathway plays an important role in the pathological mechanisms of many common human diseases. It also participates in the regulatory process of various cellular activities such as cell proliferation, metabolism, differentiation, apoptosis, cytoskeletal reorganization, and so on. It is key in the signal transduction process of growth factor receptor tyrosine kinase (RTK) and G protein‐coupled receptors.^40^, ^41^, ^42^ This signaling pathway is generally found in a disordered state in tumors. The abnormally activated signaling pathway PI3K/AKT is tightly linked to the progression of tumor cell proliferation, survival, anti‐apoptosis, metastasis, and invasion capabilities. Studies have demonstrated that signaling pathway PI3K/AKT is critical to the migration and invasion of cervical cancer. Shi et al. found that the down‐regulation of hnRNP A2/B1 in cervical cancer cells can significantly inhibit the tumor cell proliferation, migration, as well as invasion. It can block the cell cycle and induce apoptosis by means of inhibiting PI3K/AKT signaling pathway.^43^ Che et al. showed that TRIP4 promotes tumor growth and metastasis by activating MAPK, PI3K/AKT and HTERT signaling, and modulates the radiosensitivity of cervical cancer.^44^ Our study proved that by inhibiting the phosphorylation level of AKT existing in cervical cancer cells, cervical cancer cell proliferation and invasion ability are significantly reduced. The result indicated that phosphorylated AKT is one participant during the regulation process of cervical cancer cell proliferation and invasion. As a result, hsa‐mir‐133a‐2 suppresses cervical cancer progression by means of inhibiting PI3K/AKT signaling pathway mediated by LAMB3.

There were not many in vivo experiments to verify our suggestions, the regulatory effect of hsa‐mir‐133a‐2 on cervical cancer cells in vivo is still known. As a result, our study bears limitations. Moreover, the regulatory effect of hsa‐mir‐133a‐2 on other target genes throughout the development of cervical cancer as well as its expression in other malignant tumors still need to be explored.

To sum up, hsa‐mir‐133a‐2 inhibits cervical cancer cell proliferation and invasion by regulating PI3K/AKT signaling pathway mediated by LAMB3. Our study is the first study to research and evaluate the regulatory role that hsa‐mir‐133a‐2 plays in the development and progression of cervical cancer and to explore the regulatory mechanism of the hsa‐mir‐133a‐2/LAMB3 in the progression of cervical cancer preliminarily. hsa‐mir‐133a‐2 provides us a new therapeutic target for cervical cancer molecular targeted therapy.

## 5 CONCLUSION

hsa‐mir‐133a‐2 inhibits cervical cancer cell proliferation and invasion by indirectly regulating the PI3K/AKT signaling pathway, providing us with a new clinical therapy strategy for cervical cancer.

## AUTHOR CONTRIBUTIONS

**Xiaoyu Sui:** Conceptualization (equal); writing – original draft (equal); writing – review and editing (equal). **Yurong Sun:** Conceptualization (equal); resources (equal); writing – original draft (equal); writing – review and editing (equal). **Guiyu Zhang:** Conceptualization (equal); data curation (equal); writing – original draft (equal); writing – review and editing (equal). **Na Chi:** Resources (equal); writing – original draft (equal); writing – review and editing (equal). **Yitong Guan:** Project administration (equal); resources (equal); writing – original draft (equal); writing – review and editing (equal). **Dan Wang:** Data curation (equal); project administration (equal); resources (equal); writing – original draft (equal); writing – review and editing (equal). **Xiulan Li:** Data curation (equal); writing – original draft (equal); writing – review and editing (equal).

## FUNDING INFORMATION

This work was supported by the Education Department of Heilongjiang Province(2019‐KYYWF‐1247).

## CONFLICT OF INTEREST

The authors declare no conflicts of interest.

## ETHICS APPROVAL STATEMENT

The data were derived from the TargetScan database, and it was unnecessary to obtain patient consent again.

## Data Availability

Data sharing is not applicable to this article as no new data were created or analyzed in this study.
